# Partial Extraction Therapy with Early Implant Placement in the Esthetic Zone: A Clinical Case Report

**DOI:** 10.1155/2022/1045906

**Published:** 2022-09-16

**Authors:** Saleh N. Almohammed, Reem S. Abdel-Hafez, Duaa D. Ailabouni

**Affiliations:** ^1^Department of Prosthodontics, Jordan University of Science and Technology, Irbid, Jordan; ^2^Department of Preventive Dentistry, Jordan University of Science and Technology, Irbid, Jordan

## Abstract

Immediate replacement of teeth designated for extraction is an appealing treatment rationale for both the patient and the operator. However, it has been associated with a greater risk of facial recession and compromised soft-tissue esthetics. Partial extraction therapy (PET) or synonymously socket shield technique (SST) or root membrane technique (RMT) has been proposed to conserve the facial alveolar contour and soft-tissue esthetics. In this article, a special case is described where a root membrane was used to prevent the modeling of the facial aspect of the extraction socket. Partial extraction was performed allowing the socket with the facial root membrane in situ to partially heal for 8 weeks before implant placement. Successful integration and restoration were achieved with very minimal hard- and soft-tissue changes, accentuating satisfactory esthetic results as dictated by objective esthetic assessment. PET with early implant placement may be considered a viable treatment option for selected cases.

## 1. Introduction

Immediate implant placement has the advantage of reducing the overall treatment time and providing patients with expeditious replacement of extracted teeth. However, placing a dental implant in the alveolar socket does not prevent ridge dimensional changes associated with the extraction of teeth [1, 2]. The disruption of the periodontal ligament upon extraction is associated with modeling/remodeling of the alveolar bone which is considered a tooth-dependent structure [3, 4]. The facial alveolar bone is more pronouncedly affected in this resorptive process being predominantly composed of bundle bone [3, 5]. This explains the greater horizontal loss on the facial aspect compared to the lingual. These alveolar changes are also associated with soft-tissue changes that might compromise the treatment outcome and soft-tissue esthetics [3, 6].

Several studies demonstrated that immediate implant placement is associated with a greater percentage of facial recession [7, 8]; nevertheless, a minimum of intact 1 mm thickness facial plate and a thick gingival phenotype were associated with a lower risk [9]. However, these features are missing in most of the cases [10, 11], which makes it difficult to guarantee predictable esthetic outcomes.

Retaining roots was described by several authors [12, 13] in an attempt to reduce the undesirable remodeling of the alveolar bone. In 2010, Professor Hürzeler described PET (SST) for immediate implant placement based on retaining a facial root fragment at the coronal third of the alveolar socket. This was proposed to result in more favorable soft- and hard-tissue esthetics and greater patients' satisfaction [14, 15]. Beagle dog histology [14] and human histology [16] were provided as proof of bone formation between the implant surface and the root fragment. A few studies were published to demonstrate the success of this technique [17–20].

## 2. Materials and Methods

This article demonstrates the clinical and radiographic outcomes of an esthetic implant case treated utilizing the concept of PET in a modified manner, in an attempt to maintain the facial ridge contours around the installed dental implant. A descriptive report will be presented; a case with early, rather than immediate, implant placement with an overall follow-up period of 19 months. Pink and White Esthetic Scores (PES and WES) [21] were recorded to objectively assess the restoration and the related facial tissues. The patient was treated in the authors' private clinics.

## 3. The Case

**Figure 1:**
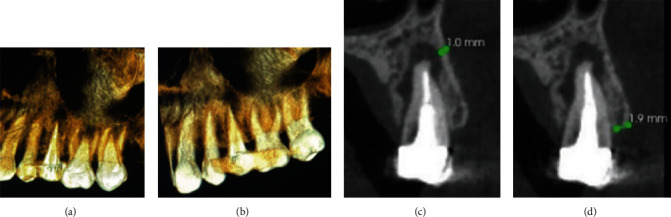
Palatal (a) and facial (b) views of M–D radiographic sections and facial plate thicknesses (c and d).

A 43-year-old medically fit female patient presented with mild chronic pain and a mobile crown of the upper left second premolar. Upon clinical and radiographic assessment, the tooth had root canal treatment and a cast-post/core with a metal-ceramic crown which had been in service for about 8 months. CBCT assessment revealed a frank periapical lesion extending close to the floor of the maxillary sinus with a facial bony plate thickness of 1.9 mm at the crest and 1.0 mm at the level of the root apex. The crown was mobile and a small manual force was enough to dislodge the postcrown leaving a root with no ferrule effect, hence designated as hopeless (Figures 1(a), 1(b), 1(c), and 1(d)). Bone sounding revealed an intact buccal plate with a normal supracrestal attachment level at the facial and interproximal areas.

**Figure 2:**
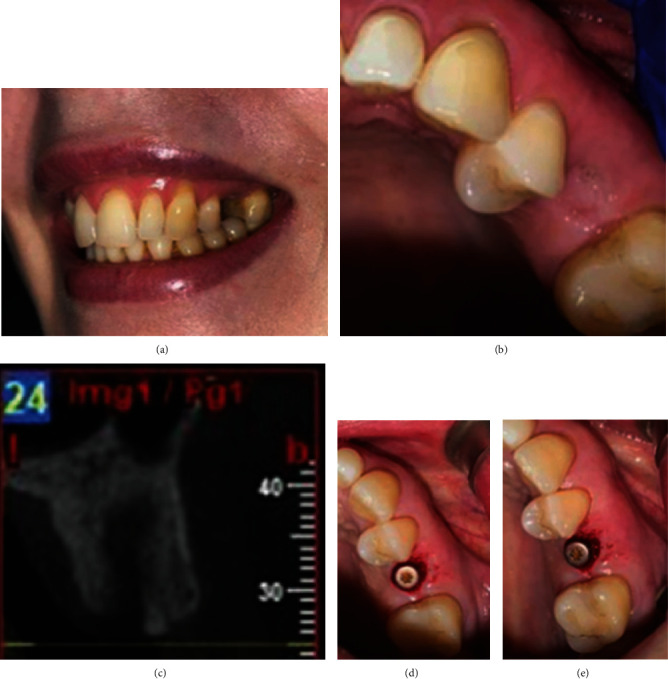
Lateral view upon smiling (a), occlusal view of extraction site of tooth 25 after 8 weeks (b), B–P radiographic section with root fragment in situ (c), and occlusal and buccal views of the healing abutment and remaining root shield immediately following implant placement (d and e).

Upon esthetic risk assessment [22], it was evident that the patient had a high lip line with a thin phenotype (Figure 2(a)), aggravated by the patient's high esthetic demands. Different treatment options were discussed with the patient and the decision was to proceed with an implant-supported restoration. The case failed to fulfill the criteria for immediate implantation as there was no sufficient apical bone to place the implant with predictable primary stability; thus, a decision to perform partial extraction of the root and wait for 4–8 weeks before implant placement [23] was adopted in an attempt to combine the benefits of both PET and early implant placement. All details were discussed with the patient and consent was obtained.

—Partial extraction and early implant placement.

After administration of the local anesthetic agent, a straight fissured carbide bur (Meisinger HM33IL/Implant burs, Surgical Carbide X-Cut Taper RA-L-010, Morris Dental, Ireland) in a surgical straight handpiece (NSK, Japan) was used to section the root in a mesiodistal direction and then section the apical portion of facial root wall, leaving a coronal fragment of the facial root wall of about 1.5–2 mm in thickness and 6–8 mm in length. This facial fragment was shortened using a flame-shaped diamond red-band bur (Jota diamond burs, Switzerland) into a “c” shape which was just coronal (about 0.5 mm) to the facial alveolar bone crest to help avoid jeopardizing the bony crest during preparation. The facial margin of the fragment was beveled to help avoid external shield exposure, and the palatal margin of the fragment was thinned and concavely shaped to avoid internal shield exposure and to provide prosthetic space. The palatal root wall and the apical part of the facial wall were extracted using periotomes and Coupland's elevators followed by debridement of the socket and disinfection using chlorhexidine gluconate 0.2%. The socket was left to heal without implant placement, socket grafting, or interim prosthesis.

After 8 weeks (Figure 2(b)), another CBCT was taken to check the root fragment and the socket healing (Figure 2(c)). It was evident that the socket had significant but incomplete bone healing with the facial root membrane stable in situ; thus, early implant placement was decided. Using the flapless soft-tissue punch technique, sequential preparation of the osteotomy was carefully performed to allow a prosthetically driven 3D implant placement (4.1 × 12mm SLActive®, Roxolid® BLT implant, Straumann®, Switzerland). To increase the amount of “press fit” and improve the implant primary stability, the last osteotomy drill (*Ø* 3.5 mm) to be used before implant placement was inserted for a depth of 7 mm rather than 12 mm and the countersinking step was skipped. The insertion torque achieved was 35 Ncm and the ISQ was 68 labiolingually and 72 mesiodistally. After that, a *Ø* 4.7 mm bottle-shaped gingival former was attached and no interim prosthesis was provided based on the patient's preference (Figures 2(d) and 2(e)). Postoperative instructions were given, and indicated medications were prescribed. This was followed by a re-evaluation visit 2 weeks after the implant placement and the patient was asked to attend after 3 months for further assessment and proceeding with the restorative phase.

—Restorative phase.

**Figure 3:**
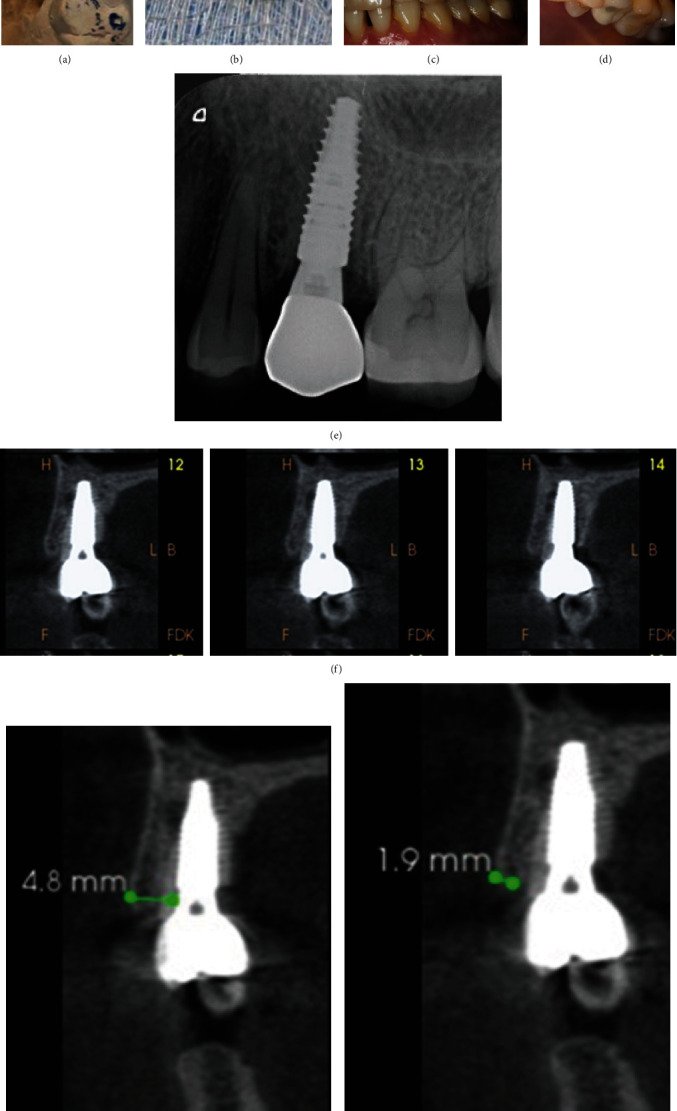
Zirconia crown cemented onto titanium abutment (a) and (b), lateral and occlusal views of crown screwed into the implant (c) and (d), postoperative PA (e), F–L radiographic sections (f), F–L section verifying width of hard facial tissues (g), and F–L section verifying width of facial plate crest (h).

After verification of clinically successful osseointegration using periapical X-ray and ISQ test (a reading of about 84); an open-tray, implant-level definitive impression was made, implant analog attached, and impression poured to fabricate a definitive working cast. A titanium abutment (Variobase® abutment, *Ø* 4.5 mm, GH = 3 mm, AH = 5.5 mm, Straumann®, Switzerland) was selected and screwed into the analog, then CAD-CAM lab technology (Ceramill map400, Ceramill motion2; Amanngirrbach, Koblach, Austria) was utilized to fabricate an occlusally vented zirconia core that precisely fits the Variobase® abutment. The dental technician built up the veneering ceramic onto the core fabricating a veneered-zirconia crown. After the laboratory work was finalized and checked on the dental cast, the definitive veneered-zirconia crown was cemented onto the abutment using dual-cured resin cement (TotalCem, ITENA, France) (Figures 3(a) and 3(b)), and then the abutment-crown assembly was transferred to the patient's mouth, tried-in and screwed into the implant using an insertion torque of 35 Ncm. Occlusal adjustment was performed, and the screw access hole was filled with composite resin, finished, and polished.

The first follow-up was performed after 2 weeks, then after 1, 3, 6, and 16 months; an overall follow-up period of 19 months since implant placement. After 16 months of loading, the PES and WES were collectively about18 of 20, indicative of an acceptable esthetic outcome with very minimal volumetric ridge changes, confirming the patient's satisfaction (Figures 3(c) and 3(d)). Also, PA and CBCT radiographs were taken; the crestal facial bony plate thickness was very similar to that before partial extraction (~1.9 mm) with a total distance of 4.8 mm from the bony crest to the implant platform. A significant bone fill between the root fragment and the implant was verified (Figures 3(e), 3(f), 3(g), and 3(h)).

## 4. Discussion

Providing patients with immediate replacement of extracted teeth with optimum soft-tissue esthetics is a desirable treatment outcome. Several techniques have been proposed to counteract the volumetric changes associated with the extraction of a tooth, such as site preservation and hard- and/or soft-tissue grafting [24, 25].

Leaving a facial portion of the root has been demonstrated to prevent or reduce the unfavorable modeling of the facial plate of bone following extraction of a tooth in what is known as PET or synonymously SST or RMT [14].

The case reported in this article provided different insights into the application of PET; the implant was placed 8 weeks following partial extraction rather than immediately.

To the extent of the authors' knowledge, in almost all reported cases of implantation using PET, implant placement was performed at the time of partial tooth extraction [19, 26]. In the case reported in this article, the decision was to prepare the root fragment and leave the socket to partially heal for 8 weeks before implant placement and without any grafting material. This protocol was selected once the assessment revealed that immediate implant placement with good primary stability was not feasible.

Successful early implant placement following soft- and hard-tissue healing was recently reported by Oliveira and his co-workers [27]. In their report, a connective tissue graft and the shield were used to preserve the socket, and the implant was placed 100 days following the extraction. In contrast, the root shield without a soft- or hard-tissue graft was used to preserve the socket in the case reported in this article. The root fragment remained stable and succeeded at maintaining the facial alveolar ridge contour during the 19-month follow-up period.

Bäumer and his co-workers in a retrospective clinical study, prepared the osteotomy through the root of the tooth to be replaced followed by the removal of the remaining root fragments except for the one on the labial aspect [19]. In contrast, in this case, the root was separated, the facial segment was prepared, and the lingual segment was extracted before the later preparation of the osteotomy. This was in an attempt to reduce the risk of dislodgment of the facial fragment and to ensure that all other parts of the root were removed.

Adequate space between the coronal edge of the shield and the subgingival contour of the crown was recommended by Gluckman et al. [26] to reduce the risk of exposure and inflammation of the root fragment. In this study, the root fragment was trimmed just coronal to the alveolar bone crest to allow for adequate soft-tissue thickness and help prevent exposure from occurring.

Different studies reported different loading protocols. Most studies provided immediate provisional restorations [19, 20, 26, 28] and a few had a delayed approach [29, 30]. In this case, the decision was to install the healing abutment and postpone restoration until osseointegration is clinically verified. This approach was adopted to allow peri-implant soft-tissue healing and supracrestal attachment formation, postulating that this might reduce the possibilities for fragment exposure.

In this case, the space between the root fragment and the implant was not filled with any biomaterial. Bäumer et al. [19], on the other hand, used an enamel matrix protein which was hypothesized to induce cementum formation and prevent resorption of the root fragment. Others reported the use of bone grafts [29]. So far, there is no evidence to support any added benefit for the use of any biomaterial.

This report and a few other reports and case series demonstrated the preliminary success of this technique in preserving the facial contour following extraction. The benefits of preserving extraction sites with less morbidity and cost cannot be emphasized enough. However, further extensive research regarding the complication rate, technique sensitivity, and longer-term follow-up is of great value to validate such a delicate and case-specific technique. Until fulfilled, PET remains a controversy.

## Data Availability

Data deposited in a repository.

## Additional Points

*Patient's Consent*. The patient signed an informed consent stating that “I declare that all the information regarding the treatment options, treatment plan, sequence of treatment, and possible complications were thoroughly discussed and approved by me before the commencement of treatment”.
